# Cannabis, lived time, and the disruption of temporal self-experience

**DOI:** 10.3389/fpsyt.2026.1921211

**Published:** 2026-10-30

**Authors:** Valerio Ricci, Domenico De Berardis, Giuseppe Maina, Giovanni Martinotti

**Affiliations:** 1 San Luigi Gonzaga Hospital, University of Turin, Turin, Italy; 2 Department of Mental Health, Psychiatric Service for Diagnosis and Treatment, Hospital “G. Mazzini”, Teramo, Italy; 3 Department of Neurosciences “Rita Levi Montalcini”, University of Turin, Turin, Italy; 4 Department of Neurosciences, Imaging and Clinical Sciences, Università degli Studi G. D’Annunzio Chieti-Pescara, Chieti, Italy

**Keywords:** cannabis, ipseity, phenomenology, self-disorder, temporality, time perception

## Abstract

Cannabis is the most widely used psychoactive substance subject to legal restriction worldwide, and one of its best-characterized acute effects is a distortion of the lived sense of time: users reliably report that time slows, fragments, or loses its ordinary continuity, an effect documented since the earliest controlled studies of marijuana intoxication and confirmed across decades of subsequent research. This paper argues that the relationship between cannabis use and psychopathology cannot be fully understood without attending to the phenomenology of this temporal disturbance. Drawing on the phenomenological tradition, on the clinical study of self-disorder through the Examination of Anomalous Self-Experience (EASE), and on the empirical literature on cannabis and time perception, we propose that cannabis functions as a temporal modulator whose disruptive action falls on an ipseity, or minimal self, that is itself temporally constituted. We treat the integration of a phenomenological vocabulary with an empirical, dose-response literature as a deliberate and contestable interpretive choice, which we make explicit rather than assume. Because cannabis is used across the lifespan and its subjective effects depend substantially on dose, frequency, expectation, and context, we discuss individual variability in temporal experience as a constraint on the model rather than a footnote to it. Adolescence and the clinical high-risk state are treated as one developmentally significant context for this disturbance. Reframing cannabis-related psychopathology as a disruption of lived temporality yields concrete, low-cost clinical implications: brief inquiry into the experience of time and the ownership of thought, EASE-informed assessment, and serial monitoring of self-disturbance.

## Introduction

Psychiatry has learned a great deal about cannabis. Its principal psychoactive constituent, Δ9-tetrahydrocannabinol (THC), acts mainly through CB1 receptors concentrated in regions long implicated in timing—the striatum, cerebellum, and thalamocortical circuitry (1), even though the plant itself contains more than a hundred cannabinoids, including CBD, whose receptor-binding profile is broader and less specific (2). This still recommends cannabis, among the very large family of substances that alter consciousness, as a comparatively tractable case for a phenomenological argument: its receptor pharmacology, dose-response relationships, and epidemiology are mapped with a confidence that the broader and still-expanding class of new psychoactive substances cannot match, since many of its members have psychopharmacology that remains only partially characterized (3). Grouping such heterogeneous compounds together for a phenomenological claim risks the same error as writing about “medicines” as though aspirin and chemotherapy shared a mechanism. A single, well-studied substance is the more defensible unit of analysis, and we restrict our argument to it.

And yet, for all that is known about how cannabis acts on the brain, one dimension of the experience it produces has received comparatively little integration into clinical practice: time. Not time as a pharmacokinetic curve, but time as it is lived—the texture of experience from one moment to the next. Cannabis is unusually well documented on this point. Altered time perception is among the most consistently reported subjective effects of intoxication, observed since the first controlled studies of marijuana (4, 5) and confirmed across decades of subsequent laboratory research using time estimation, production, and reproduction paradigms (1, 6).

This omission matters, because phenomenological psychiatry has insisted for a century that temporality is not a backdrop to mental life but its scaffolding (7–9). Inner time, on this view, is not a clock running quietly in the background. It is the thread that ties the just-past, the vivid now, and the anticipated next into a single continuous experience—the thread that lets a self feel like one self, persisting, rather than a series of disconnected flashes. Cut it, as happens in psychosis or in severe dissociation, and what breaks is not merely the sense of clock-time but the self. Phenomenologists call this fracture an ipseity disturbance (10, 11).

Our thesis is that cannabis use intersects with two pressures, not one. The first is pharmacological and immediate: a substance whose acute action reliably includes an alteration of temporal experience—an effect that is not unique to cannabis, since comparable distortions are reported with stimulants and psychedelics (12), but one that is unusually well documented in the cannabis-specific literature, however heterogeneous parts of that literature remain (13**).** The second is clinical: those who go on to develop psychotic disorders after cannabis exposure often have an ipseity that was already fragile, whatever their age. We treat adolescence and the clinical high-risk (CHR) state as a developmentally important instance of this fragility, because the temporal self is still consolidating across the second decade of life (14, 15) and because most longitudinal psychosis-risk research has, in fact, been conducted in that age group. But cannabis use is not the preserve of the young. Its prevalence among adults over fifty has risen sharply in the past decade, driven by changing attitudes, legalization, and self-medication for pain, sleep, and mood (16), and clinicians should be wary of the long-standing tendency in media and policy discourse to read the risks of a substance primarily through the figure of the wayward adolescent who requires correction (17). What follows about developmental vulnerability is a claim about one window in which temporal disruption carries particular weight, not a claim that the disruption, or its psychiatric significance, belongs to the young alone.

This paper builds and defends a framework rather than systematically reviewing a body of evidence, developing it in step with what it implies for assessment and care, on the premise that an insight unusable at the bedside has failed its purpose.

## Phenomenology and empirical psychopharmacology: a note on method

This paper deliberately mixes two registers that do not naturally sit together: a phenomenological vocabulary, built to describe invariant structures of experience independent of their cause, and an empirical, dose-response vocabulary, built to describe how a specific compound changes specific outcomes. We do not think this mixture is illegitimate, but we think it needs defending rather than assuming, because the two traditions answer different questions and a careless blend can make a substance look like it “causes” a disturbance in the strong sense phenomenology was designed to bracket.

Our license for the mixture is narrow and specific. When an exogenous, dose-titratable compound with a well-characterized, if not exclusive, receptor profile (18) reliably produces a particular class of subjective report—time dilation, looping repetition, a feeling of watching oneself from outside—across naive and experienced users, in laboratory and naturalistic settings, that report becomes a reasonable explanandum for phenomenological description, and the convergence of the two literatures has clinical precedent: the cognitive dysmetria account of schizophrenia and the broader literature on lived time in psychosis already combine first-person description with circuit-level neurobiology (1, 19). What the mixture cannot license is the inference, made too quickly in places, that cannabis simply causes a fixed disturbance of ipseity, as though pulling a single causal lever produced a single determinate effect. The pathway from THC to the experiences we describe runs through dose, frequency and history of use, expectation, and context—a dependency taken up directly in the section on individual variability below. Read every claim in this paper of the form “cannabis disrupts ipseity” as shorthand for a hypothesis under construction, anchored in a real and replicated subjective effect, not as an established causal mechanism.

## Inner time and the self under construction

Husserl’s analysis of inner time-consciousness (8) is the place to begin. Every moment of experience, he argued, is not a single point but a small chord of three notes sounding together: retention, the phase that has just slipped past but still echoes within consciousness; primal impression, the live edge of the now; and protention, the faint anticipation of what is about to come. These overlap and together form one flowing present, the living present. It is this structure that lets us hear a melody as a melody rather than a string of unrelated tones—and that underlies the slow assembly of a personal identity, the binding of one’s own moments into a story that holds together over time.

This structure is not static across the lifespan. The capacity to integrate experience into a continuous biography, and to inhabit an extended future as one’s own, develops markedly across adolescence, in step with the maturation of the prefrontal cortex and of the networks supporting thought about oneself and others (14, 15), which is one reason the adolescent self is unusually plastic and, in some respects, unusually exposed. It does not follow that the same temporal scaffolding is irrelevant once that maturation is complete; adults of any age depend on the same retention-protention structure to feel like one continuous self, and a substance that bends that structure does so whenever it is used, not only in youth.

Minkowski took the phenomenology of time into the clinic. In Lived Time (9) he argued that the core of schizophrenic experience was not delusion or hallucination but something quieter and more fundamental: a breakdown of temporal flow. The patient does not merely read the clock wrong. What fails is the “vital contact with reality”—the living pull of the future that, in health, keeps drawing us forward. Sever it, and time congeals. The future stops beckoning, and the person is left marooned in a present that has gone static, strange, no longer quite alive.

A century later, these ideas have been given clinical teeth. The Examination of Anomalous Self-Experience, or EASE (20), is a structured interview probing disturbances of self across five domains. Two matter especially here. The first reaches directly into temporal experience—thought interference, thought blockage, loss of thought ipseity, disruptions of the flow of consciousness. The second reaches into the minimal self, including that unsettling sense that one’s own thoughts, feelings, and perceptions have lost their “mineness,” their Meinheit—that they are no longer quite one’s own.

It is worth being precise about ipseity, because the word does a great deal of work in what follows (21). It does not refer to the autobiographical self, the “I” with a name and a history and a story to tell. It refers to something more basic and more silent: the bare, pre-reflective sense that experience is happening to someone, that it is mine before I ever stop to reflect that it is mine. Disturbances of ipseity are now treated as a possible marker of vulnerability to psychosis, detectable in clinical high-risk individuals—often, though not only, adolescents and young adults—well before any frank symptom appears (22, 23).

Here is the hinge of the whole argument. Ipseity is itself made of time. The minimal self is not a fixed point but a moving process—exactly the retention-to-protention arc Husserl described. So any substance that bends the arc, that speeds it, slows it, or fragments it, is doing something far more intimate than altering mood or perception: it is, in a strict phenomenological sense, disturbing ipseity. That is the bridge from pharmacology to psychosis. And the bridge has a practical end. A clinician who wants to catch ipseity disturbance early in a cannabis-using patient, whatever their age, does not need a laboratory or an hour of testing—only a few honest questions. Has time felt different during and after use: faster, slower, somehow broken? Have your own thoughts begun to feel as though they were happening to you rather than being thought by you? Have you felt strangely outside yourself, watching from a distance? These cost minutes and map almost one-to-one onto the temporal and ipseity items of the EASE, yet in everyday practice they are almost never asked.

## Cannabis as a temporal modulator

Among cannabis’s many subjective effects, the alteration of time perception is among the oldest documented and most reliably replicated. The first controlled study to measure it objectively gave THC to marijuana-naive volunteers and found dose-dependent changes in time estimation in the range of seconds to minutes (5); a companion study the same decade found that naive and experienced users showed measurably different patterns of “temporal disintegration” under the drug (4). Decades of subsequent work—using time estimation, time production, and time reproduction tasks—confirm that THC reliably overestimates the duration of short intervals and accelerates the felt pace of subjective time, with effects that depend on dose and on the user’s history with the drug (1, 6). Clinical case material from the same era described an acute syndrome of “temporal disintegration and depersonalization” during marihuana intoxication—the felt continuity between past, present, and future loosening, accompanied by a sense of detachment from one’s own thoughts and body (24, 25).

Seen through a phenomenological lens, this body of evidence describes a single underlying disturbance with two faces. At low to moderate doses and in naive users, cannabis tends to dilate and thicken subjective time: the present stretches, intervals are overestimated, and intoxication itself is frequently sought out precisely for this slowing, a kind of temporal immersion that can feel like relief from an otherwise rushed existence. At higher doses, with heavier use, or in vulnerable individuals, the same dilation can tip into fragmentation: thoughts that loop and repeat, a déjà-vu quality to experience, the feeling of being stuck fast in a present that will not move (1, 26). A plausible bridge between dilation and fragmentation, consistent with the Husserlian model introduced earlier, is that once the retentional phase is prolonged beyond its ordinary span, a content of thought fails to recede into the just-past and continues to be processed within the living present as though still current; phenomenologically, this is what would present as a thought that loops or repeats rather than simply passing. In EASE terms these are losses of thought ipseity and disturbances of the flow of consciousness. The clinically important point is easy to miss: looping and dilation are unremarkable during intoxication; what should make a clinician pay attention is persistence after the drug has cleared, and especially a patient’s report that the looping thoughts do not feel entirely his or her own.

The mechanistic story now has some texture. Dopaminergic and cerebellar circuits implicated in interval timing are densely populated with CB1 receptors, and chronic cannabis exposure is associated with disruption of the cerebello-thalamo-cortical circuit that supports both motor and cognitive timing (1). Chronic use also appears to leave lasting alterations in cerebellum-dependent learning that outlast intoxication (27), and at the network level, cannabis disrupts the default mode network tied to self-referential processing and the integration of experience across time (27). None of this is confined to the acute state. The signal worth tracking over months is not any specific distortion of clock time but the slow emergence of a trait-like disturbance of mine-ness—the patient’s growing report that thoughts, feelings, and perceptions no longer feel securely his or her own. That, more than anything, marks the road toward frank psychosis, and the kinship between persisting cannabis-related temporal fragmentation and severe depersonalization is well recognized (28). It is worth noting that depersonalization, on Simeon’s own account, is not exhausted by a disturbance of thought ownership; it carries a marked perceptual component as well, affecting the felt reality of the body and of the external world, so that its overlap with the temporal-ipseity model proposed here should be read as partial rather than complete.

## Individual variability: dose, history, expectation, and the limits of attribution

The literature just summarized is also, on close reading, a literature about difference rather than uniformity, and this needs stating plainly rather than folded into a footnote. The earliest controlled studies already found that naive and experienced users respond differently to the same dose of THC (4), and the most careful modern replication found that frequent cannabis users show a substantially blunted time-perception response to intravenous THC relative to infrequent users and non-users, whose time estimation and production are altered at every dose tested (6). Tolerance, in other words, is not incidental to the phenomenon; it is part of what the phenomenon is. A model that treats cannabis as a temporal modulator with one characteristic signature, independent of who is using it and how often, would be contradicted by the data it claims to explain.

A second source of variability sits outside pharmacology altogether. Since at least the mid-twentieth century, researchers studying psychoactive drugs have distinguished the substance itself from the user’s “set”—personality, expectation, mood, and intention—and the “setting” in which it is taken, and have shown that these non-pharmacological parameters substantially shape the qualitative character of the experience, sometimes as much as the drug itself (29). A user who already expects, from cultural lore or prior experience, that cannabis “slows time down” may be primed to notice and report exactly that, independent of any underlying change in temporal cognition; conversely, a first-time or unsuspecting user may experience the same intoxication as frightening derealization rather than pleasant dilation. We do not know how much of the temporal phenomenology reported in clinical interviews reflects a stable change in self-experience versus a learned interpretive script applied to an ambiguous bodily state, and the honest position is that both probably contribute in proportions that vary across individuals and occasions.

Large-scale analyses of first-person accounts bear this out. A recent computational study of more than thirty-six thousand user-submitted experience reports on the harm-reduction archive Erowid.org found that the qualitative character of a drug experience—positive, frightening, dissociative, transcendent—depends heavily on context, dose, and individual user variables, and is poorly predicted by substance identity alone (30). This is a caution against the move, common in this literature and risked by our own argument above, of treating a substance as though it stamped a fixed phenomenological signature onto whoever takes it. The same person, on different occasions, with different intentions, in different company, can have qualitatively different experiences of the same drug at the same dose; and a user well versed in the expected effects of cannabis is in a different epistemic position than a naive user encountering an unfamiliar bodily state for the first time, simply by virtue of having a ready-made attribution available.

This matters for the present argument in two ways. First, any claim that cannabis “disrupts ipseity” must be read as a claim about a population-level tendency under specified conditions of dose and use history, not a deterministic prediction for any given patient; the same caution that applies to (novel psychoactive substances) NPS as a heterogeneous class applies, in a smaller way, even within a single well-characterized substance. Second, and less often discussed, we do not know whether attributing a transient temporal anomaly to “the drug” rather than to oneself is protective—externalizing an unsettling experience and limiting its psychological uptake—or whether it instead normalizes anomalous experience and delays the recognition that something durable is changing. Both processes are plausible, and to our knowledge neither has been tracked longitudinally. We flag this explicitly as an open question rather than resolve it by assumption, because the clinical implications of the two possibilities point in different directions.

## A vulnerability model, qualified by variability

Pulling these threads together, we propose a model in three parts. It links a pharmacological action with strong empirical support, a phenomenological account of what that action targets, and an explicit acknowledgment of the individual variability that moderates both. This is a heuristic, meant to generate hypotheses and organize what we see, not a finished mechanism.

The first level is pharmacological. Cannabis enters as a compound whose well-replicated acute effect is to alter the dilation, continuity, or fragmentation of subjective time, an effect with a known dose-response relationship and a known dependency on use history (6). In the short run, for many users, this can feel like relief—a borrowed restoration of agency over one’s own time, sought deliberately in stressful or overstimulating circumstances, whatever the user’s age.

The second level is phenomenological. Repeated heavy use, particularly in those already biologically or developmentally vulnerable, appears in some cases to corrode the temporal structures that intoxication initially modulated for relief. The mechanisms run on two tracks: a biological one, involving chronic CB1 dysregulation and disruption of cerebellar and default-mode circuitry (1, 27), and a phenomenological one, in which repeated pharmacological disruption of the retention-protention structure may progressively degrade a person’s own capacity to constitute temporal continuity, independent of the drug’s continued use. At some threshold, crossed faster in vulnerable individuals, this erosion can tip into frank psychosis, itself readable as the self’s last, catastrophic effort to force experience back into temporal order (31).

The third level is the moderating one, and it is here that the model differs most from a simple causal story. Set, setting, dose, frequency, and expectation determine which of the trajectories above a given episode of use is likely to follow (6, 29). Developmental stage is one such moderator, and an important one: CHR adolescents have an ipseity that is already attenuated, the minimal self already fragile, the retention-to-protention arc already a little frayed (32), so that cannabis’s temporal effects land on already-compromised ground. But it is one moderator among several, not the master variable, and the same model should in principle apply—with different weights on dose history, expectation, and biological vulnerability—to an older adult self-medicating chronic pain or insomnia with high-potency cannabis products whose effects on a less plastic but not invulnerable temporal self remain comparatively understudied (16).

## Implications for assessment, services, and prevention

If the model holds, even in this qualified form, its sharpest consequences are for how we assess people who use cannabis heavily, particularly those at clinical or ultra-high risk for psychosis, regardless of age. The tools we rely on—the CAARMS, the SIPS, the BSABS—capture attenuated positive symptoms, cognitive disorganization, and functional decline (33, 34) but were not built to reach the phenomenological core of ipseity and temporal disturbance that the EASE captures (20). Folding the EASE, or at least its items on the stream of consciousness and ipseity, into routine assessment of heavy cannabis users would help identify those in whom use has begun eroding the infrastructure of the self. This is not abstract: anomalous self-experiences, and temporal and ipseity disturbances in particular, are far more prevalent in psychotic disorders than in controls (35).

Because the erosion, where it occurs, is progressive rather than fixed (36), one assessment is rarely enough. Brief, repeated inquiry into how time feels and whether thoughts feel owned can show which way things are moving: fading, which is reassuring, or hardening into something trait-like, which should prompt closer monitoring. Given the variability documented above, this inquiry should routinely include questions about dose, frequency, expectation, and the context of use, since the same report of “time slowing down” means something different in a naive user’s first experience than in a daily user’s tenth year of heavy consumption.

Two brief composite vignettes convey this better than any list of criteria, and we offer one at each end of the age range the model is meant to cover. Picture a sixteen-year-old who has used high-potency cannabis bought from a peer. The acute episode has passed, yet he returns still saying that time will not move, that he gets caught on a thought that loops, and—the detail that should stop you—that the looping thoughts do not feel like his. A standard examination might note adolescent anxiety and send him back to school; the temporal-phenomenological reading recognizes a loss of thought ipseity persisting beyond intoxication and treats it as an attenuated self-disorder warranting EASE-informed assessment and follow-up. Picture, too, a sixty-three-year-old who began using cannabis edibles nightly for chronic pain after retirement. Over several months she notices that evenings feel strangely elongated even off the drug, and that she sometimes watches herself going through the motions of dinner “from a small distance.” With no psychiatric history and no obvious stressor, she may be reassured that this is simply what cannabis does, whereas the same framework asks whether a persisting depersonalization is consolidating in a person whose risk profile we understand far less well than an adolescent’s, precisely because so much of the field’s attention has gone to the young. Both vignettes are composite, with no evidentiary weight; they show only how the lens changes what a clinician notices, and in whom.

The therapeutic implication is unavoidable: intervention cannot stop at the pharmacological management of the acute episode. If temporal alienation, however it arose, feeds both the use and the vulnerability it breeds, care must reach the existential dimension of time. Phenomenologically oriented and existential psychotherapy, and mindfulness understood as temporal re-integration—a non-reactive presence within the living present (37)—are theoretically well matched to this picture, whatever the patient’s age.

Concretely, phenomenologically oriented psychotherapy would proceed less by symptom checklist than by careful, non-leading exploration of how the patient’s own sense of continuity across past, present, and future has changed, using the same register of questioning that underlies the EASE itself. Existential approaches would address the patient’s relation to finitude and to an anticipated future that intoxication, and its aftermath, can flatten or foreclose. Mindfulness-based practice, on this reading, is not primarily relaxation training but a structured cultivation of attention to the living present, in which retention and protention are held lightly rather than collapsed or set looping, and could in principle be adapted specifically toward the temporal and ipseity disturbances described here rather than deployed as a generic adjunct. We offer these as directions consistent with the framework rather than as therapies whose efficacy for this particular disturbance has been established; to our knowledge, none has yet been tested against ipseity or temporal-disturbance outcomes specifically in cannabis-using populations.

Beyond the clinic, the framework outlined here also points toward a more experimental avenue. Human laboratory studies administering THC under controlled, ethically reviewed conditions could pair standard time-estimation and time-reproduction paradigms with structured, EASE-informed phenomenological interviewing within the same dosing session, so that a single study yields both a quantitative measure of temporal distortion and a first-person account of how ipseity is experienced while it is occurring. Such designs remain uncommon, and we raise the possibility cautiously, aware that pairing structured self-report instruments with acute drug administration carries its own methodological and ethical complexities; but if the phenomenological and empirical registers mixed in this paper are to be integrated rather than only juxtaposed, human laboratory pharmacology is arguably where that integration would first need to be tested.

## Limitations

This is a speculative, programmatic argument, not an empirical demonstration: a opinion paper articulates a framework that cannot yet be tested directly. Narrowing the focus to cannabis trades breadth for tractability, and we accept that trade explicitly; the model says nothing reliable about other substances, and extending it would require redoing much of this argument from the pharmacology up. The mixture of phenomenological and empirical vocabularies, defended above, remains philosophically contested, and our license for it is narrower than the rhetoric of “cannabis disrupts ipseity” sometimes suggests; the phenomenological tradition’s relation to causal, dose-response accounts of the self remains genuinely open (38). The developmental claims, though grounded in established work on adolescent brain maturation, are used to motivate the framework, not to test it, and we have tried, not entirely successfully, to keep adolescence one window among others rather than the paper’s true subject in disguise. The variability we describe—dose, frequency, expectation, set and setting—is real and substantial, and we have not modeled it formally; we have only insisted that any causal-sounding claim in this paper be read with that variability in mind. The EASE was validated chiefly in schizophrenia-spectrum rather than substance-using populations (39, 40). The clinical vignettes offered above are composite and carry no evidentiary weight.

## Conclusions

We cannot grasp why heavy cannabis use is associated with such a wide range of psychiatric outcomes without a phenomenological account of what happens when time comes apart, and we cannot give that account responsibly without acknowledging how much the experience varies with dose, history, expectation, and circumstance. Cannabis is, on the evidence summarized here, a genuine modulator of lived temporality, not merely of mood or perception, but its action is conditional rather than fixed, and it falls on selves at very different stages of construction—adolescent and adult, naive and experienced. Those most at risk of cannabis-associated psychosis are plausibly those whose hold on their own time was already tenuous, whatever their age; for them, use is both a symptom of an existing vulnerability and, potentially, an engine that drives it deeper. The clinical payoff is concrete and inexpensive. A few plain questions—how does time feel, do your thoughts still feel like yours, how often and how much do you use, what did you expect to feel—asked routinely of any patient using cannabis heavily can surface attenuated self-disorders that standard examination misses; the EASE can formalize them, and serial inquiry can show whether they fade or consolidate.

## Data Availability

The original contributions presented in the study are included in the article/supplementary material, further inquiries can be directed to the corresponding author/s.
